# Wheat differential gene expression induced by different races of *Puccinia triticina*

**DOI:** 10.1371/journal.pone.0198350

**Published:** 2018-06-07

**Authors:** Kerri A. Neugebauer, Myron Bruce, Tim Todd, Harold N. Trick, John P. Fellers

**Affiliations:** 1 Department of Plant Pathology, Kansas State University, Manhattan, KS, United States of America; 2 USDA- ARS, Hard Winter Wheat Genetics Research Unit, Manhattan, KS, United States of America; University of Nebraska-Lincoln, UNITED STATES

## Abstract

*Puccinia triticina*, the causal agent of wheat leaf rust, causes significant losses in wheat yield and quality each year worldwide. During leaf rust infection, the host plant recognizes numerous molecules, some of which trigger host defenses. Although *P*. *triticina* reproduces clonally, there is still variation within the population due to a high mutation frequency, host specificity, and environmental adaptation. This study explores how wheat responds on a gene expression level to different *P*. *triticina* races. Six *P*. *triticina* races were inoculated onto a susceptible wheat variety and samples were taken at six days post inoculation, just prior to pustule eruption. RNA sequence data identified 63 wheat genes differentially expressed between the six races. A time course, conducted over the first seven days post inoculation, was used to examine the expression pattern of 63 genes during infection. Forty-seven wheat genes were verified to have differential expression. Three common expression patterns were identified. In addition, two genes were associated with race specific gene expression. Differential expression of an ER molecular chaperone gene was associated with races from two different *P*. *triticina* lineages. Also, differential expression in an alanine glyoxylate aminotransferase gene was associated with races with virulence shifts for leaf rust resistance genes.

## Introduction

The struggle between fungi and their host plants is an evolutionary battleground. Pathogenic fungi must have a means of overcoming host defenses in order to obtain nutrients and complete their life cycle. Fungi have specialized effector molecules, which are used to combat plant defenses and reprogram host cells. However, plants have several layers of defenses. The first being pathogen-associated molecular pattern (PAMP)-triggered immunity (PTI; [1]). Pathogen recognition receptors (PRR) span the host plasma membrane and detect PAMPs triggering an accumulation of reactive oxygen species, increased ethylene production, and eventually an induction of the salicylic acid pathway [2]. The next layer is direct or indirect recognition of specific pathogen effectors by nucleotide binding, leucine rich repeat (NB-LRR) containing proteins starting a cascade of events leading to a more intense form of resistance, called effector triggered immunity (ETI; [1]). The hallmark of ETI is the localization of infection by a hypersensitive response that results in programmed cell death [3]. The ETI interaction between effectors and NB-LRR resistance genes work in a gene-for-gene manor [4] and is the basis of most of the “major gene” genetic resistance in crop improvement.

Wheat (*Triticum aestivum* L.) endures yield losses from a wide range of biotic stresses including many diverse fungal pathogens such as *Puccinia triticina* Eriks, the causal agent of wheat leaf rust or brown rust. Leaf rust is widespread and infects wheat worldwide causing significant losses. Annual worldwide losses due to leaf rust have been estimated at $2 billion [5] and from 2000 to 2004, yield losses in the United States were estimated at over $350 million [6]. *P*. *triticina* has one of the most complex lifecycles of any fungal pathogen with five different spore types on two unrelated hosts. The alternate host, meadow rue, (*Thalictrum speciosissimum* L.) is required for the completion of the sexual phase of the life cycle, however, it is not native to North America. Thus, leaf rust reproduces asexually and infects wheat via urediniospores in North America [6–8].

Each year, 70+ different races are collected in North America [6–8]. Even without a sexual stage, the fungus has an inherent, yet unknown, mutation mechanism to produce races that overcome NB-LRR based ETI. Using markers or whole genome sequencing, each North American race can be linked to one of six major clonal linages, but can be uniquely identified by a combination of phenotypic reactions in response to a set of differential isogenic R gene wheat lines and molecular markers [9–10]. Genetic resistance is the most commonly utilized yield loss prevention strategy for leaf rust [8]. Genetic resistance can be categorized as either major or minor gene resistance. Most major genes provide high levels of race-specific resistance and encode NB-LRR proteins [11]. Because of its race specific mechanism, major gene resistance induces heavy selection pressure for virulence shifts in the population [6–7], leading to the breakdown of major resistance genes within 4–5 years after release [12].

Effectors are very important to the fungus and elimination, mutation, or lack of wild type expression may have a significant fitness penalty to the fungus [13]. However, leaf rust is often able to survive after the ETI-recognized effector has mutated, suggesting the protein function has not changed or is functionally redundant. However, there is an exception suggesting a survivability cost in leaf rust. Though the effector is not known, plant breeders have noticed emerging races of *P*. *triticina* overcoming *Lr16* will tend to disappear from the population once *Lr16* is removed from wheat cultivars (Kolmer, personal communication). Most of the avirulence effectors, however, appear to not have a negative effect on the fungus, when a change in the effector has been selected for by a wheat variety. A hypothesis was developed to test what effector changes do to the plant, in the absence of resistance genes, and whether wheat gene expression responses can be detected which are associated with a particular race or lineage. The following research was aimed at testing the hypothesis by evaluating wheat gene expression of a single susceptible cultivar, when exposed to six different *P*. *triticina* races from two different lineages.

## Materials and methods

### Plant growth conditions

All inoculations used the hard red spring wheat (*Triticum aestivum* L.), leaf rust susceptible cultivar Thatcher (PI 168659, University of Minnesota, 1936). Thatcher seedlings were grown in 20 cm x 20 cm square pans containing Metro Mix 360 soil medium (SunGro, Vancouver, Canada) and maintained in a Percival 30-B growth chamber (Percival Scientific, Perry IA) at 18°C with 16 h day/ 8 h night cycles. Seedlings were inoculated at the two to three leaf-stage. Five mg/ml of fresh or desiccated spores were suspended in Soltrol 170 isoparaffin (Philips 66, Bartlesville, OK) and inoculated using an atomizer and compressed air at 40 PSI. Mock (control) inoculations were treated the same with Soltrol, but without spores. Plants were incubated in Percival I-36DL dew chamber overnight for 16 hours, 100% humidity, and 18°C, then returned to a growth chamber as above. Time scale sampling, fixing, staining with Uvitex-2B, and imaging of infected tissue used the techniques developed by our colleagues [14]

### *P*. *triticina* races

Six races of *P*. *triticina* were obtained from Dr. Jim Kolmer (USDA-ARS Cereal Disease Laboratory, St. Paul, MN), two from North American lineage 3 (NA3): MHDS and MLDS, and four from North American lineage 5 (NA5): MJBJ, TDBG, THBJ, and TNRJ [10]. Avirulence and virulence definitions for each race are listed in Table 1. All races used are virulent on Thatcher and have a 3+ infection type on the 0–4 Stakman rating scale [15–16]. The *P*. *triticina* urediniospores were stored at -80°C and heat shocked at 40°C for 10 minutes prior to inoculation.

**Table 1:** *P*. *triticina* races used in the research and their definitions based on reactions to the Thatcher *Lr* isogenic differential set. Top line with each race represents the *Lr* genes the race is avirulent to, while the bottom line represents the *Lr* genes the race is virulent to.

Race	Avirulent to: Virulent to:
MHDS	*Lr2a*, *Lr2c*, *Lr9*, *Lr24*, *Lr3ka*, *Lr11*, *Lr30*, *Lr18* ***Lr1*, *Lr3a*, *Lr16*, *Lr26*, *Lr17*, *LrB*, *Lr10*, *Lr14a***
MLDS	*Lr2a*, *Lr2c*, *Lr16*, *Lr24*, *Lr26*, *Lr3ka*, *Lr11*, *Lr30*, *Lr18* ***Lr1*, *Lr3a*, *Lr9*, *Lr17*, *LrB*, *Lr10*, *Lr14a***
MJBJ	*Lr2a*, *Lr2c*, *Lr9*, *Lr26*, *Lr3ka*, *Lr11*, *Lr17*, *Lr30*, *LrB*, *Lr18* ***Lr1*, *Lr3a*, *Lr16*, *Lr24*, *Lr10*, *Lr14a***
TDBG	*Lr9*, *Lr16*, *Lr26*, *Lr3ka*, *Lr11*, *Lr17*, *Lr30*, *LrB*, *Lr14a*, *Lr18* ***Lr1*, *Lr2a*, *Lr2c*, *Lr3a*, *Lr10*, *Lr24***
THBJ	*Lr9*, *Lr24*, *Lr3ka*, *Lr11*, *Lr17*, *Lr30*, *LrB*, *Lr18* ***Lr1*, *Lr2a*, *Lr2c*, *Lr3a*, *Lr16*, *Lr26*, *Lr10*, *Lr14a***
TNRJ	*Lr16*, *Lr26*, *Lr17*, *LrB*, *Lr18* ***Lr1*, *Lr2a*, *Lr2c*, *Lr3a*, *Lr9*, *Lr24*, *Lr3ka*, *Lr11*, *Lr30*, *Lr10*, *Lr14a***

### RNA isolation and sequencing

Leaf tissue was isolated from inoculated leaves 6 days post inoculation (DPI). One leaf, measuring 2.5 cm, was taken from five independent plants inoculated with the same race, pooled, and immediately frozen with liquid N_2_. Total RNA was isolated from each pooled sample and processed using the *mir*Vana miRNA isolation kit (AM1560, RNA Life Technologies, Carlsbad, CA) according to the manufacturer’s protocol to obtain total RNA. RNA was quantified using a Nanodrop ND1000 spectrophotometer (Thermo Fisher Scientific, Waltham, MA) and sent to Cofactor Genomics (St. Louis, MO) for RNAseq analysis, assembly, and primary analysis. The RNA was sequenced by Cofactor Genomics' in-house protocols [17].

### Unigene set and expression analysis

A wheat unigene set was developed by first assembling cDNA reads from the oil only Thatcher control tissue at 6 DPI. cDNA was assembled into contigs using Trinity v2011059 [18] using reads with 80% of the bases with Q scores > 20. Contigs were combined with the TIGR *Triticum* cDNA unigene set to form a combined reference unigene assembly of 33,055 ESTs. Read counts were made by aligning Illumina reads against this reference set using novoalign v2.06.09 (Novocraft, Selangor, Malaysia). Analysis of expression and Boolean comparisons were made using ActiveSite (Cofactor Genomics, St. Louis, MO) with thresholds of > 2-fold expression difference and a minimum total read count of > 90 for each mRNA fragment across each treatment.

### Expression profiling and quantitative real time-PCR (qRT-PCR) analysis

A time course study was performed using the same six races and inoculation procedures described above. Biological samples consisted of single 2.5 cm leaf segments of heavily infected leaves from five plants. Three pooled samples (biological reps) were made representing 15 total plants. Leaf tissue was collected at 7 time points: 0, 1, 2, 3, 4, 5, and 6 DPI, flash frozen in liquid N_2,_ and stored at -80°C. Samples of two controls, oil only and non-inoculated plants were made at the same time. Total RNA and quantification methods were as before. First strand cDNA was synthesized with Superscript II (Thermo Fisher, Waltham, MA) according to the manufacturer’s recommendations using one μg total RNA and 50 ng random hexamers as primers.

qRT-PCR primers were designed using MacVector V12.7.3 (MacVector, Cary, NC) and Primer Quest (Integrated DNA Technologies, Coralville, IA) from the assembled contigs with the parameters of: T_m_ 58°C, GC content ~50%, 18 to 24 nt, and 100–250 bp amplicon size. Efficiency of all primers was determined to be between 90% and 110% on a five point dilution series from 1X cDNA concentration to 0.0005. All reactions were performed in the Bio-Rad CFX96 Real-Time System (Hercules, CA) using the Bio-Rad iQ SYBR Green Supermix in a 25 μl reaction which contained 6 μl cDNA template (diluted in a 2:1 ratio), and 10 pmol of each primer. The qRT-PCR conditions were as follows: 95°C for 3 minutes, 40 cycles of 95°C for 10 seconds, and 62°C for 30 seconds. The run was completed with a melt curve: 65°C to 95°C heating in 0.5°C increments for 5 seconds. Three biological and three technical replicates were obtained for all reactions. Relative expression was calculated using the ΔΔC_T_ method [19]. The cycle threshold (C_T_) values were averaged and compared to the C_T_ values of the Soltrol inoculated control. The resulting C_T_ value was subtracted from the C_T_ value of the internal wheat ubiquitin (UBQ [20]) housekeeping gene. (GOI: gene of interest) Formula are as follows:

ΔCTGOI=CToil−CTtreatment

ΔCTUBQ=CToil−CTtreatment

ΔΔCT=ΔCTGOI−ΔCTUBQ

### Statistical analysis

A repeated measures analysis of *Puccinia triticina* race and time effects on wheat gene expression was conducted using SAS Proc Mixed (SAS Institute Inc., Cary, NC, USA). Fungal lineage, race nested within lineage, time effects and interactions were treated as fixed effects, with biological replicate treated as a random effect. Heterogeneous compound symmetry was selected as the covariance structure, with model fit evaluated by Akaike information criteria (AIC) and residual pattern. Slice effects of race nested within lineage, as well as linear contrasts of the effects of “M” vs. “T” races on wheat gene expression pattern were also evaluated.

## Results

### RNA expression analysis

**Fig 1:**
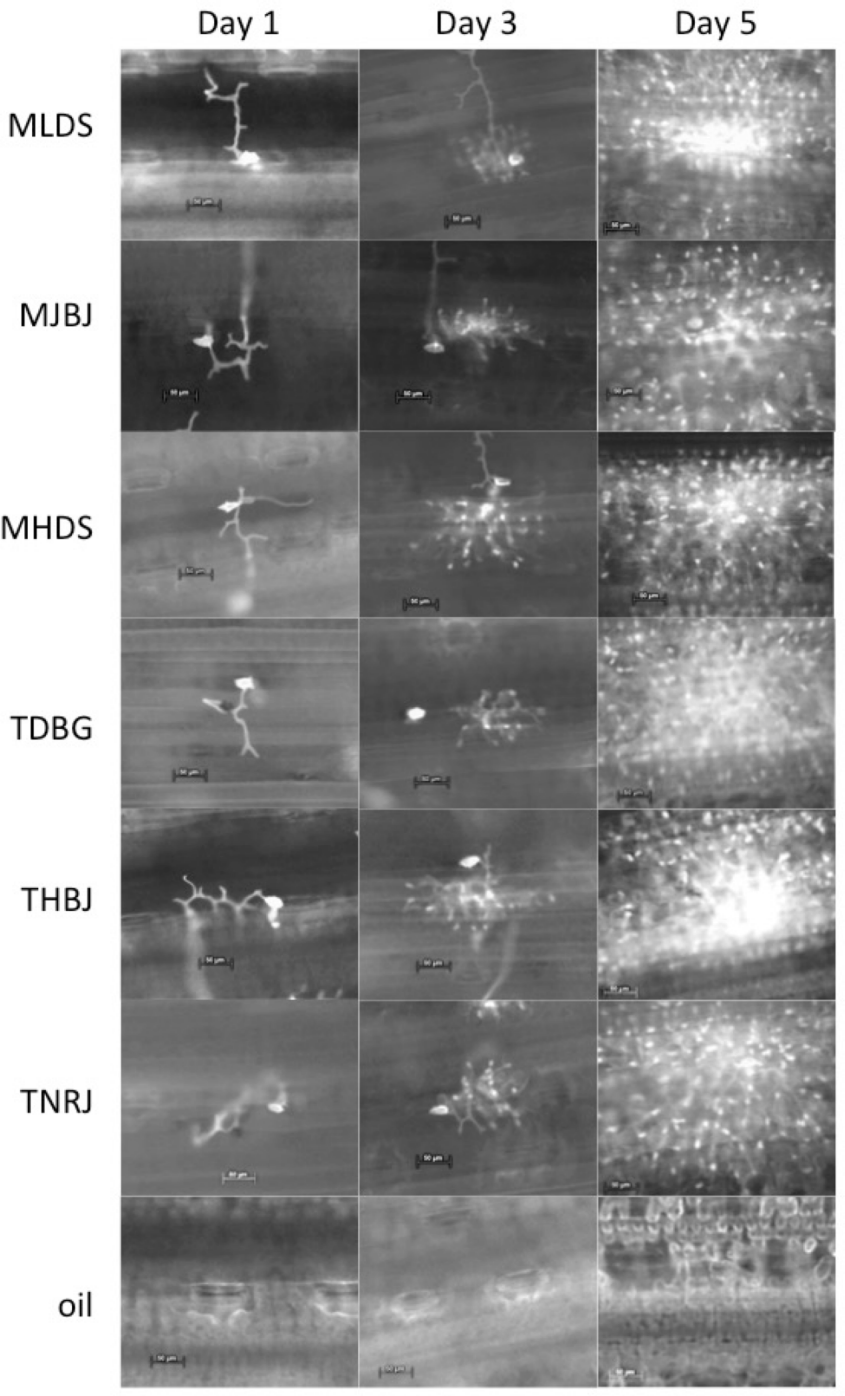
*P*. *triticina* growth and development in a compatible interaction of six races of *P*. *triticina* and a no fungus-Soltrol only control on the susceptible variety, Thatcher, at 1, 3, and 5 DPI. At day 1, spores have germinated, found a stomata, formed appressorium, and started growth inside the leaf. Day 3 and 5 show fungal development within the leaf tissue. Fungal structures are stained with Uvitex 2B and size bars represent 50 microns.

The six chosen *P*. *triticina* races are commonly found in North America and range in their virulence to leaf rust resistance genes. Each race was inoculated onto Thatcher. Symptoms were not visually present until 3 DPI, consisting of small chlorotic focal regions along the leaf. Microscopic analysis (Fig 1) determined that each fungal race was developing at a similar rate. By 3 DPI, fungal structures had spread within the leaf tissue and by 5 DPI, the hyphae were well established (Fig 1). By 6 DPI, fungal pustules were clearly formed, however, urediniospores were sub-dermal and had not erupted. Regions of heavy infection had chlorotic regions between the pustules. At 6 DPI, the fungus has also begun secondary growth and pustule formation and thus many of the infective fungal structures are present in the wheat tissue.

To reduce plant-to-plant variability and simplify the workflow, a pooling strategy was used in the initial RNA sequencing. Using a single leaf from 5 plants, tissue was pooled for RNA isolation. Each tissue sample generated between 23.4 to 33.2 million, 60 bp paired-end reads, of which 43.4–56.7% aligned to the wheat EST reference (S1 Table). The tissue samples also contained fungal transcripts and a summary of those results were reported previously [17]. ActiveSite analysis is based on normalized read counts for a specific EST reference and was used to make all possible comparisons between the six treatments. A total of 63 mRNA fragments were selected that met the above requirements (S2 Table). The functions of the 63 candidates were grouped into major functions based on homologies: energy and metabolism, membrane function and protein transport, stress-related proteins, RNA binding proteins, secondary metabolism, repeat elements, and unknown function.

### Plant response to infection

**Fig 2:**
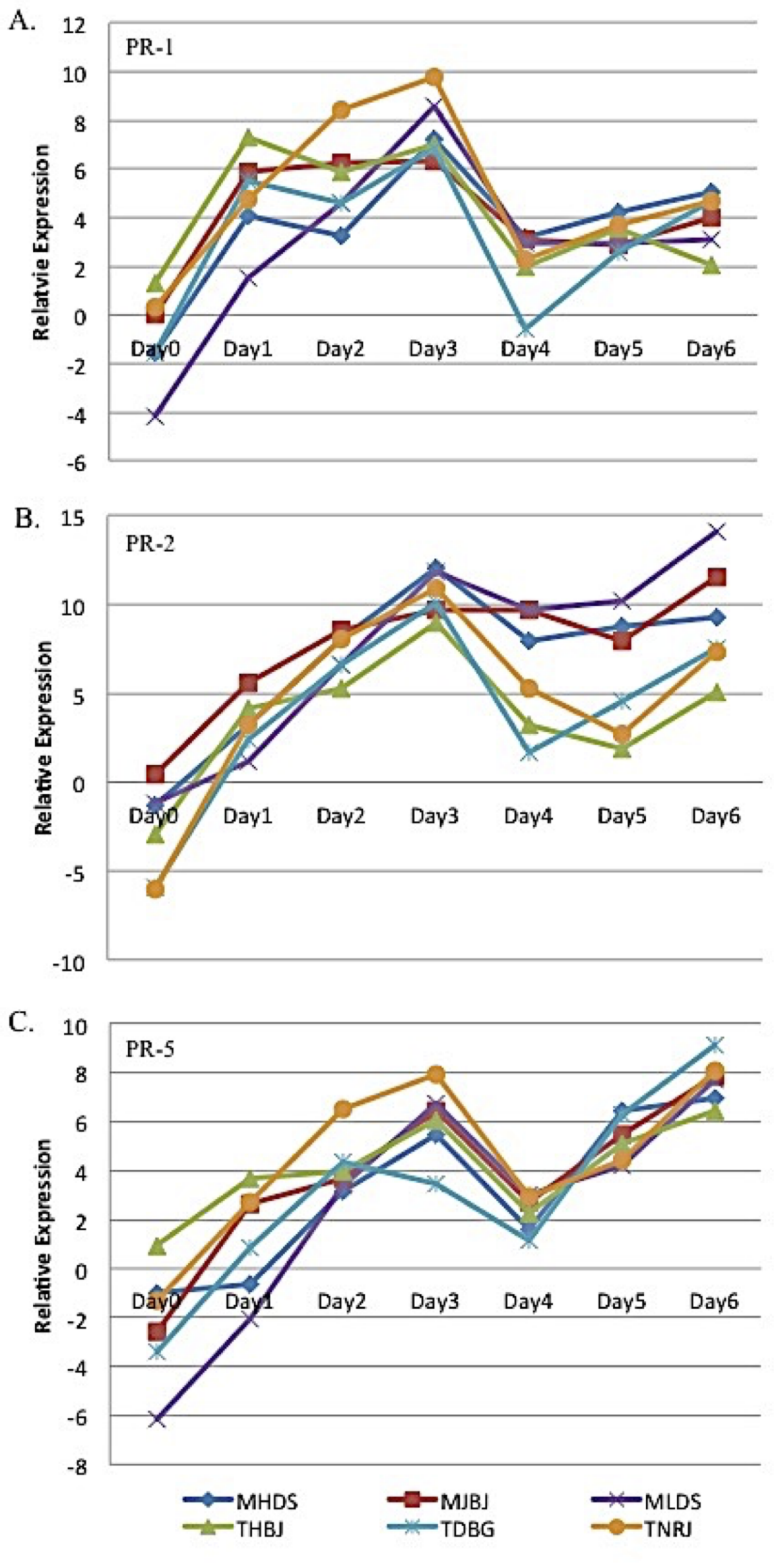
Gene expression of three wheat pathogen response (PR) genes, (A) *PR-1* (FJ815167); (B) *PR-2 β-1*,*3 glucanase* (DQ090946), and (C) *PR-5* thaumatin-like protein (AF384146) in response to six races of *P*. *triticina*.

qRT-PCR was used to verify the differential expression of the candidate transcripts. Primers were designed (S3 Table) to the cDNA fragments and due to the elimination of candidates aligning to retroelements and the lack of amplification from some of the primer combinations, the candidates were narrowed to 61. In addition to the selected gene candidates, the expression of three pathogen response (PR) proteins, PR-1 (FJ815167), PR-2 beta 1,3 glucanase (DQ090946), and PR-5 thaumatin-like protein (AF384146), were also evaluated to characterize PAMP response gene expression (Fig 2). Expression for all three PR genes followed an expected pattern. Little or no expression at day 0, peaking at 3 DPI, decreasing at 4 DPI, and in the cases of PR-2 and PR-5, increasing again to 3 DPI levels by 6 DPI. Interestingly, "M" races induced an approximately 3–5 fold higher expression of PR-2 at 5 DPI than "T" races (Fig 2B).

**Fig 3:**
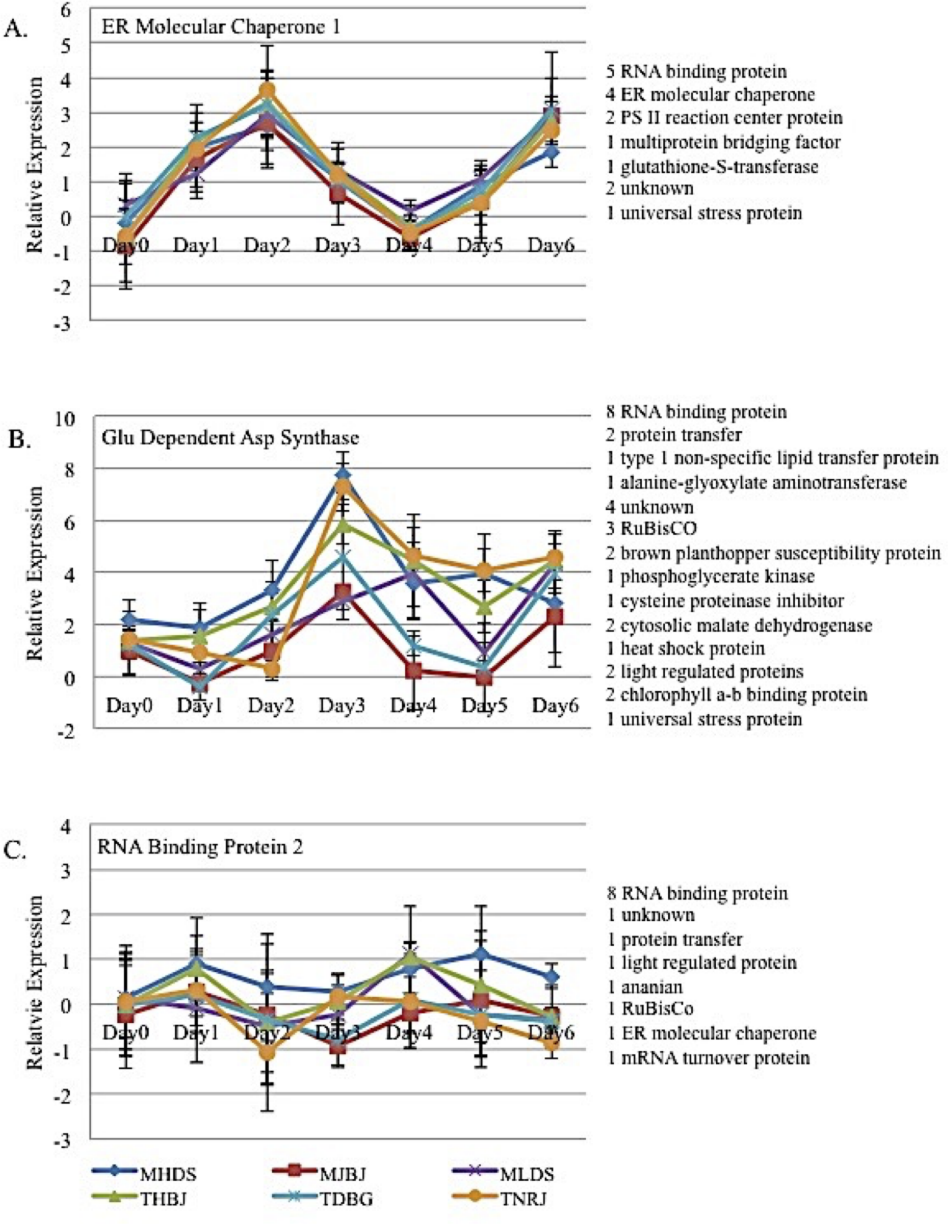
Temporal patterns of wheat genes originally identified as being differential expressed at six days post inoculation. A) Wheat gene expression is similar in response to all six races. B) The wheat gene expression is varied in response to race, but a clear pattern cannot be determined. C) The wheat gene does not appear to be induced by *P*. *triticina* infection. The errors bars show the standard error.

qRT-PCR verified the two-fold differential gene expression at 6 DPI for 47 of the 63 gene candidates and provided information on the gene response to infection (GenBank accessions JZ976938-JZ976982). There were three groups of temporal expression patterns among the 47 gene candidates. First, 16 of the genes followed a PTI-like gene expression pattern similar to that of an ER molecular chaperone (KC894716 [21]; contig number 16207, JZ976938), in which gene expression peaked at 2 DPI, dropped to nearly 0 at day 4 then increased again at 6 DPI (Fig 3A). There was no significant variance in gene expression due to race treatments for this gene (Table 2). Sixteen genes were found to exhibit this expression pattern, including five RNA binding proteins, four ER molecular chaperones, two photosystem II reaction center proteins, one multiprotein bridging factor, one glutathione-S-transferase, two genes with unknown function, and one universal stress protein.

**Table 2:** Analysis of variance for *P*. *triticina* lineage and race effects on expression of selected wheat genes over 6 days post inoculation.

	Genes													
	ER Chaperone				GluDAS**		RNA Binding Protein						AlaAT***	
	16207*		16209		2283		13984		15148		20213		955	
Fixed effects	*F* value	*P*	*F* value	*P*	*F* value	*P*	*F* value	*P*	*F* value	*P*	*F* value	*P*	*F* value	*P*
Lineage	0.02	0.9021	3.42	0.068	3.36	0.0704	5.53	**0.0211**	4.97	**0.0286**	0.71	0.4023	3.44	0.0671
Race (lineage)	0.20	0.9389	0.10	0.9829	11.39	**< .0001**	2.21	0.0747	1.64	0.1712	0.08	0.9875	0.56	0.69
Day	22.12	**< .0001**	10.43	**< .0001**	22.08	**< .0001**	2.23	**0.0478**	12.94	**< .0001**	62.99	**< .0001**	12.63	**< .0001**
Lineage × day	0.88	0.5134	1.48	0.1956	0.30	0.9341	0.33	0.9213	0.59	0.7386	1.74	0.1215	1.81	0.1062
Race (Lineage × day)	0.25	0.9998	0.38	0.9954	1.57	0.0683	0.48	0.9788	0.52	0.9653	0.63	0.8986	0.67	0.8632
Slice effects:														
Race (NA5)	0.18	0.911	0.12	0.9497	11.81	**< .0001**	1.32	0.2736	1.17	0.3251	0.05	0.9851	0.75	0.5259
Race (NA3)	0.26	0.6139	0.04	0.8431	10.14	**0.0021**	4.89	**0.0298**	3.05	0.0843	0.18	0.6714	0.01	0.9419
Contrast:	*t* value	*P*	*t* value	*P*	*t* value	*P*	*t* value	*P*	*t* value	*P*	*t* value	*P*	*t* value	*P*
M vs. T races	-0.38	0.7039	1.46	0.1492	-2.07	**0.0414**	1.27	0.2086	1.00	0.3202	0.35	0.7305	-2.36	**0.0205**

* Unigene contig number, also associated with primer number in S3 Table.

** Glutamine dependent asparagine synthetase (Fig 3B).

**Fig 4:**
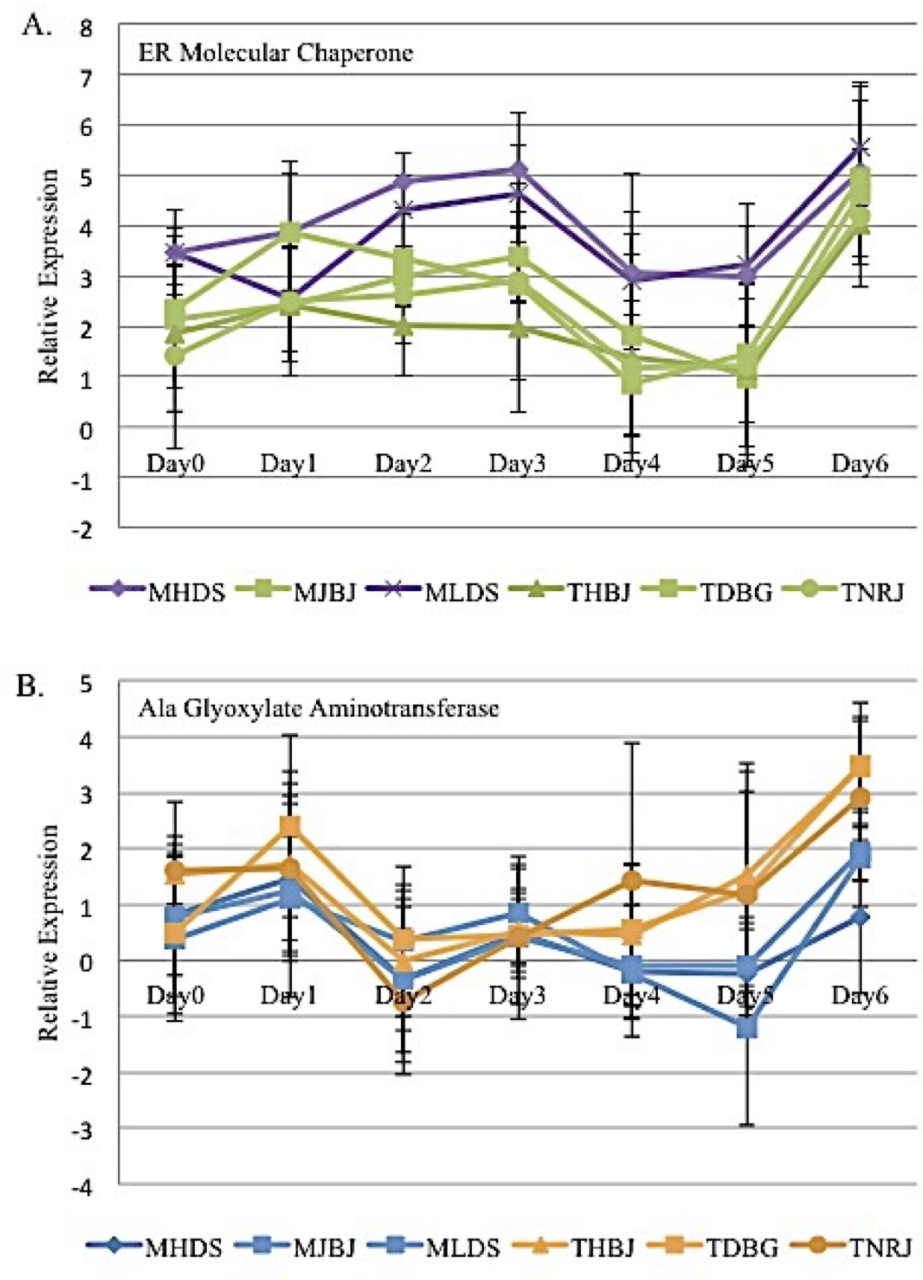
Wheat genes with differential expression associated with (A) lineage and (B) race. The errors bars show the standard error.

*** Alanine glyoxylate aminotransferase (Fig 4B).

The second group has a general pattern of having a low gene expression at day 0, then increasing at 3–4 DPI (Fig 3B). In Fig 3B, a glutamine dependent asparagine synthetase (GluDAS AY621539 [22]; contig number 2283, JZ976945) expressed highly at day 3, but with some races, the gene was not expressing on days 4 and 5, while expression was high with the other races. Gene expression varied with race exposure, but lineage and “M” vs. “T” comparisons explained relatively little of the variation (Table 2). Thirty-one genes fit this expression pattern including eight RNA binding proteins, two protein transfer, one type one non-specific lipid transfer protein, one alanine-glyoxylate aminotransferase, four unknowns, three RuBisCo, two brown plant hopper susceptibility proteins, one phosphoglycerate kinase, one cysteine proteinase inhibitor, two cytosolic malate dehydrogenase, one heat shock protein, two light regulated proteins, two chlorophyll a-b binding proteins, one universal stress protein, and one glutamine dependent asparagine synthetase.

The remaining 15 genes of the 61 showed little or no expression change during the six day test period (Fig 3C), even though they were identified in the primary expression analysis as having differences to the controls at day 6. The example in Fig 3C is an RNA binding protein (contig number 13984, JZ976977), which showed minimal variation across days, but, as was observed for GluDAS, a race effect that was unrelated to lineage or “M” vs. “T” groupings (Table 2). Fifteen genes exhibited this expression pattern including ten RNA binding proteins, one gene of unknown function, one protein transfer, one light regulated protein, one ananain cysteine-type protease, one RuBisCo, one ER molecular chaperone, and one mRNA turnover protein.

### Lineage and “M” vs. “T” race responses

The major question of this work was to determine if plant gene responses could be detected which are associated with races and/or lineages. Isolates used in this study belong to two of the six lineages in North America [10]. MHDS and MLDS are in lineage NA3, while MJBJ, THBJ, TDBG, and TNRJ are in lineage NA5. No gene displayed a clear effect that could be associated with lineage alone, although a molecular chaperone (contig number 16209;) did display a gene expression pattern that suggested a putative lineage effect (Fig 4A; Table 2). There was evidence that “M” races and "T" races were associated with differential expression (*P* = 0.0205, Table 2) of an alanine glyoxylate aminotransferase (AlaAT, KD512071; contig number 955, JZ976940). AlaAT showed 1.5-fold higher expression in response to “T” races than to “M” races at 3 DPI through 6 DPI (Fig 4B) and was not being expressed at 4–5 DPI with the “M” race treatment.

## Discussion

Before characterizing mRNA fragments that were differentially expressed during *P*. *triticina* infection, the expression of three PR proteins, PR-1, PR-2, and PR-5, were evaluated in response to *P*. *triticina* infection. PR proteins are induced in a PTI response to a wide variety of pathogens and are also involved in plant development. Specifically, PR-1, PR-2, and PR-5 have been shown to inhibit growth of a variety of fungi [23]. PR-2 proteins have a β-1,3- glucanase activity [24] and are induced in the presence of fungi that contain β-1,3-glucans in their cell walls [23] while PR-5 functions as a thaumatin-like protein [25–27]. The specific function of PR-1 is still unknown [28]. The initial expression patterns of the PR genes indicated that the PTI pathway has been activated (Fig 2). From 0 DPI through 3 DPI, the appressorium is formed, enters into the host, comes in contact with the cell wall, and forms haustoria (Fig 1, Day 3). Between day 3 and 4, the fungus is transitioning between growth and spore formation and beginning secondary growth. The gene expression of PR-2 had the same general trend as PR-1 and PR-5, but “M” and “T” races induced differential expression at 4–6 DPI. This suggests a common factor between these two groups may be inducing the differing responses.

There have been numerous previous studies that have evaluated wheat gene expression in wheat rust compatible reactions. cDNA AFLPs were used to identify transcript-derived fragments (TDF) that were differentially expressed throughout the first 7 d of *Puccinia striiformis* f. sp. *tritici* infection [29]. RuBisCo and chlorophyll a-b binding protein were found to be down-regulated during the early stages of infection and identified ten up-regulated TDFs that were associated with signal transduction functions [29]. Using an Affymetrix Wheat GeneChip, 73 transcripts were induced by *Puccinia striiformis* f. sp. *tritici* in a compatible reaction [30]. Transcript accumulation peaked at 24 h after infection. Of the transcripts found, 25 transcripts were defense-related, six were involved in signal transduction, eight were involved in metabolism, seven transcripts were in protein and carbohydrate transport, 19 were specific to biotrophic interactions, four had functions related to electron transport, and 25 transcripts were of unknown function [30]. In addition, 42 probe sets were identified being up regulated and one probe set that was repressed in a compatible *Puccinia striiformis* f. sp. *tritici* wheat interaction [31]. The majority of the probe sets had functions related to plant defense, while nine of the probe sets functioned in carbohydrate metabolism [31]. *De novo* transcriptome assemblies were used to identify host genes differentially expressed in an *Lr28* resistant reaction and in a compatible leaf rust interaction [32]. Reactive oxygen species enzymes and glutathione-S-transferase genes increased leading to lower the oxidation state in infected susceptible plants compared to resistant. Similar to the present study, annotated sequences associated with lipid metabolism, carbon metabolism, and RNA transport pathways were found [32].

S2 Table shows similar expression levels of RNA contigs aligned with wheat genes having similar proposed function. Wheat is a hexaploid with an assumed triple redundancy of each gene. Multiple contigs are likely due to assembly and small nucleotide changes in conserved homologs in the three genomes of wheat. The pathogen can be using or affecting one or all of the host genes during the biotrophic interaction. In an RNA expression study, each read can be aligned very specifically to a particular target, and that target can be used to design specific primers for quantitative PCR. In this study, each contig identified had specific primers designed, but it was not determined whether the primers used were specific to homologs on the A, B, or D genome of wheat. Another consideration is the fungus. *P*. *triticina* is dikaryotic with two genomes. In North America, *P*. *triticina* populations are clonal, asexual, and have high linkage disequilibrium [10,33]. Changes in an effector are mostly seen in isolates that act as heterozygous [34–36], but may actually have differences in redundancy.

Genes encoding low temperature responsive and glycine-rich RNA binding proteins made up 35% of the total genes examined and had a range of gene expression patterns. All of the sequences for RNA binding proteins aligned to different segments of one RNA binding protein (AGI04359) and could imply specificity in the wheat-*P*. *triticina* interaction. RNA-binding proteins (RBP) are a group of regulatory factors interacting with the binding domains of single-stranded or double-stranded RNA throughout all post-transcriptional processes including: mRNA splicing, polyadenlyation, sequence editing, transport, mRNA stability, mRNA localization, RNA export, chromatin modification, and translation [37–39]. In addition, stress activated RNA binding proteins may function as molecular chaperones and assist in the translation of stress-associated genes to help plants recover from cellular stress injuries [37,40]. RNA binding proteins have also been reported to be involved in plant pathogen interactions and may help regulate the plant defense system [37,41]. The *Pseudomonas syringae* effector protein, *HopU1*, modified *Arabidopsis* RNA-binding proteins during infection and as a result, the RBPs had a reduced ability to bind and regulate their target RNAs, which caused increased susceptibility [37]. In barley, two glycine-rich RNA binding proteins, *Hvgrp2* and *Hvgrp3*, displayed increased mRNA levels in the presence of fungal pathogens *Erysiphe graminis* and *Rhynchosporium secalis* in incompatible and compatible interactions [42].

Endoplasmic reticulum (ER) molecular chaperones were the proposed function of five of the characterized mRNA fragments. The sequences of the five mRNA fragments aligned to different segments of the same ER molecular chaperone (KC894716 [20]). There were two ER molecular chaperones with particularly interesting gene expression determined by real time PCR. The expression level of ER molecular chaperone-1 in response to all six races was the same, but the expression changed drastically every other day (Fig 3A). It is thought that the expression of this gene is being influenced by the pathogen during the first week of infection and therefore may be essential for successful infection of *P*. *triticina*. In contrast, the expression of ER molecular chaperone-2 provided evidence of association with different lineages (Fig 4A). ER molecular chaperones are involved in the post-translational processing of proteins. Almost all secreted proteins enter the endoplasmic reticulum (ER) during or immediately following synthesis. When the proteins enter the ER, the ER molecular chaperones recognize mis-folded or unstable proteins and aid in correcting their orientation upon exit of the ER. Correct protein folding and maturation in the ER is essential for protein transport in the secretory pathway [43].

An alanine-glyoxylate aminotransferase (contig number 955) showed differential race specific expression during the first week of infection between "M" and "T" races. Alanine aminotransferase (AlaAT) belongs to a pyridoxal phosphate multigene family and functions in animals, plants, yeast, and bacteria. AlaAT is an enzyme that catalyzes the transfer of an amino group from glutamate to pyruvate forming 2-oxoglutarate and alanine [44–45]. AlaAT is thought to be involved in many physiological processes throughout the life cycle of plants. AlaAT regulation has been associated with responses to low-oxygen stress, carbon stress, and nitrogen stress in many plant species. For example, AlaAT was induced during hypoxia in barley, maize, soybean, and *Arabidopsis* [45] and is necessary for seed and seedling germination of *Medicago truncatula* in hypoxia conditions [44]. Although AlaAT has not been previously characterized in response to pathogens, it may be functioning as a stress response to *P*. *triticina* infection. *P*. *triticina* could induce carbon or nitrogen stress in the host as the pathogen accumulates plant nutrients for its own growth. One of the common differences between "M" and "T" races is the avirulence to virulence shift on *Lr2A* and *Lr2C*. However, until the effectors are identified, it can only be speculated that the effector has an effect on AlaAT.

### Conclusions

In order to stay ahead in the arms race between *P*. *triticina* and wheat, a greater understanding of the interaction between the host and pathogen is needed. This study aimed to identify wheat genes whose expression was affected by *P*. *triticina* and to characterize the expression of these genes during the first week of infection. A total of 63 wheat genes having differential expression were identified. The gene expression of 61 of the wheat genes was further evaluated with a time course study using real time PCR. Two wheat genes with suggested race specific expression were identified providing evidence that the variance in *P*. *triticina* effector repertoires leads to different wheat interactions. In an experiment like this, fungal development differences could be responsible for the differences seen. However, Fig 1 shows that each of the races developed at the same rate, and the statistical design with replications, controls, and internal reaction controls, are strong enough to make these conclusions. This could provide much needed insight into the wheat-*P*. *triticina* interaction and the role pathogen effectors play in infection. Many of the wheat genes with similar expression in response to multiple races could be essential for *P*. *triticina* infection. Both groups of genes need further study and characterization to demonstrate these findings *in planta*.

## Supporting information

S1 Table Sequencing and alignment summary of wheat cDNA using 60 bp paired-end Illumina sequencing platform. Each sample represents wheat seedling tissue 6 DPI with indicated *P*. *triticina* race. cDNA was aligned to a wheat EST singleton reference.
(DOCX)

S2 Table ActiveSite summary of initial RNA expression analysis. **Searches were based on all Boolean combinations of expression ratios of > 2 and expression sums > 90**. Expression is based on normalized read counts of alignments to a *Triticum aestivum* EST unigene reference set.
(DOCX)

S3 Table Primer sequences for real-time PCR primers. The primer number in the name column corresponds to the cDNA contig made from assembled wheat cDNAs. The “F” is the forward primer and the “R” is the reverse primer of the primer pair.
(DOCX)
