# Supplementary material for: Wheat differential gene expression induced by different races of Puccinia triticina
Source: PLoS One. 2018 Jun 7;13(6):e0198350. doi: 10.1371/journal.pone.0198350 (PMC5991701; doi:10.1371/journal.pone.0198350)
Supplement: S1 Table — Each sample represents wheat seedling tissue 6 DPI with indicated P. triticina race. cDNA was aligned to a wheat EST singleton reference. (DOCX) [file pone.0198350.s001.docx]

**S1 Table**. **Sequencing and alignment summary of wheat cDNA using 60 bp paired-end Illumina sequencing platform**. Each sample represents wheat seedling tissue 6 DPI with indicated *P. triticina* race. cDNA was aligned to a wheat EST singleton reference.

| Race used for Inoculation | Total Wheat  Reads | Aligned Reads to Wheat Reference | Wheat Aligned  Base Pairs |
| --- | --- | --- | --- |
| MLDS | 25,556,420 | 11,758,504 (46.0%) | 1,411,020,480 |
| MHDS | 26,419,162 | 11,466,255 (43.4%) | 1,375,950,600 |
| MJBJ | 23,415,788 | 11,105,663 (47.4%) | 1,332,679,560 |
| TDBG | 27,731,985 | 13,660,759 (49.3%) | 1,639,291,080 |
| THBJ | 33,225,893 | 18,754,321 (56.4%) | 2,250,518,520 |
| TNRJ | 28,404,510 | 16,118,395 (56.7%) | 1,934,207,400 |
