# Supplementary material for: Wheat differential gene expression induced by different races of Puccinia triticina
Source: PLoS One. 2018 Jun 7;13(6):e0198350. doi: 10.1371/journal.pone.0198350 (PMC5991701; doi:10.1371/journal.pone.0198350)
Supplement: S2 Table — Searches were based on all Boolean combinations of expression ratios of > 2 and expression sums > 90. Expression is based on normalized read counts of alignments to a Triticum aestivum EST unigene reference set. (DOCX) [file pone.0198350.s002.docx]

**S2 Table**.  **ActiveSite summary of initial RNA expression analysis. Searches were based on all Boolean combinations of expression ratios of > 2 and expression sums > 90**. Expression is based on normalized read counts of alignments to a *Triticum aestivum* EST unigene reference set.

| Unigene* | Boolean§ | Log  Ratio | MLDS | MHDS | MJBJ | TDBG | THBJ | TNRJ | Sum | BLASTX annotation |
| --- | --- | --- | --- | --- | --- | --- | --- | --- | --- | --- |
| 2935:13-635 | 000001 | 3.50 | 19.78 | 43.31 | 8.46 | 19.31 | 6.94 | 78.51 | 176.31 | chlorophyll a/b binding |
| 2947:24-1052 | 000001 | 3.34 | 31.11 | 71.14 | 8.45 | 20.06 | 11.46 | 85.74 | 227.96 | chlorophyll a/b binding |
| 38:1-577 | 000001 | 3.61 | 35.05 | 60.55 | 10.03 | 27.32 | 11.76 | 122.2 | 266.91 | chlorophyll a/b binding |
| 4231:1-744 | 000001 | 3.40 | 8.77 | 18.43 | 5.46 | 17.95 | 10.93 | 57.83 | 119.37 | uncharacterized |
| 4596:25-1121 | 000100 | 3.35 | 11.31 | 26.31 | 36.83 | 86.14 | 20.59 | 8.42 | 189.6 | hydroxyphenylpyruvate dioxygenase |
| 6778:993-1290 | 000100 | 3.49 | 25.41 | 66.39 | 57.26 | 84.22 | 7.04 | 11.2 | 251.52 | xylanase inhibitor |
| 955:1-1266 | 000100 | 3.72 | 5.69 | 10.74 | 31.46 | 74.97 | 43.01 | 11.37 | 177.24 | acetyl ornithine aminotransferase |
| 16780:1-270 | 001000 | 3.59 | 14.7 | 19.29 | 30.9 | 15.59 | 7.48 | 2.13 | 90.09 | multiprotein bridging factor |
| 4010:33-792 | 001000 | 4.44 | 16.16 | 31.65 | 98.19 | 9.73 | 7.42 | 4.53 | 167.68 | SCP exrtracellular protein |
| 1892:314-567 | 010000 | 4.56 | 31.88 | 112.52 | 12.76 | 42.12 | 4.43 | 5.51 | 209.22 | light induced protein |
| 1911:15-229 | 010000 | 4.19 | 79.49 | 224.58 | 25.39 | 83.37 | 11.37 | 16.09 | 440.29 | light regulated protein |
| 3318:20-235 | 010000 | 4.01 | 75.18 | 153.51 | 18.07 | 76.87 | 9.5 | 11.31 | 344.44 | light regulated protein |
| 959:60-334 | 010000 | 4.22 | 34.06 | 79.38 | 17.67 | 40.68 | 6.77 | 3.59 | 182.15 | unknown |
| 12282:56-163 | 010001 | 4.95 | 11.78 | 60.32 | 3.89 | 11.89 | 1.8 | 20.9 | 110.58 | chlorophyll a/b binding |
| 26293:47-744 | 010001 | 4.85 | 12.89 | 39.95 | 4.81 | 11.85 | 1.28 | 20.71 | 91.49 | chlorophyll a/b binding |
| 26320:9-437 | 010001 | 4.34 | 13.93 | 39.36 | 7.97 | 8.19 | 1.81 | 26.39 | 97.65 | chlorophyll a/b binding |
| 3423:73-1240 | 010001 | 4.06 | 26.07 | 86.55 | 6.63 | 23.48 | 9.66 | 110.95 | 263.34 | chlorophyll a/b binding |
| 3426:5-1144 | 010001 | 3.63 | 85.74 | 181.23 | 41 | 74.67 | 14.68 | 177.03 | 574.35 | chlorophyll a/b binding |
| 3457:101-410 | 010001 | 4.46 | 14.94 | 58.88 | 3.86 | 28.24 | 15.01 | 84.66 | 205.59 | chlorophyll a/b binding |
| 4360:7-306 | 010001 | 5.04 | 17.46 | 31.05 | 5.98 | 7.76 | 1.68 | 50.1 | 114.03 | chlorophyll a/b binding |
| 13973:14-232 | 010100 | 5.04 | 17.4 | 59.05 | 5.21 | 21.71 | 2.83 | 1.5 | 107.7 | RNA binding protein |
| 1519:229-525 | 010100 | 4.29 | 29.4 | 64.46 | 14.94 | 35.66 | 6.66 | 2.77 | 153.89 | unknown |
| 1891:399-773 | 010100 | 4.88 | 26.96 | 88.55 | 13.6 | 32.61 | 2.79 | 4.5 | 169.01 | light regulated protein |
| 5652:408-503 | 010100 | 3.43 | 14.33 | 40.02 | 32.5 | 41.78 | 3.63 | 15.86 | 148.12 | a-amylase inhibitor |
| 777:225-494 | 010100 | 5.06 | 17.05 | 60.22 | 12.2 | 23.12 | 5.75 | 1.52 | 119.86 | unknown |
| 16209:326-864 | 011000 | 3.72 | 24.53 | 55.15 | 44.97 | 30.42 | 3.89 | 17.96 | 176.92 | heat shock protein |
| 12547:1-278 | 100000 | 3.37 | 110.15 | 91.2 | 27.14 | 48.46 | 10.17 | 35.24 | 322.36 | lipid transfer protein, |
| 1801:20-339 | 100000 | 3.77 | 224.38 | 160.45 | 32.01 | 87.93 | 16.49 | 28.2 | 549.46 | unknown |
| 24701:3-290 | 100000 | 3.34 | 77.5 | 56.09 | 15 | 23.87 | 7.28 | 9.15 | 188.89 | stress protein family |
| 3159:39-382 | 100000 | 3.52 | 83.81 | 52.26 | 14.21 | 30.65 | 7.33 | 12.05 | 200.31 | unknown |
| 3429:8633-9943 | 100000 | 3.74 | 334.94 | 249.8 | 47.97 | 113.42 | 25.15 | 34.06 | 805.34 | retroelement |
| 3503:1363-2988 | 100000 | 3.78 | 268.55 | 165.86 | 34.67 | 90.07 | 19.49 | 20.37 | 599.01 | cell surface protein |
| 3575:7-6696 | 100000 | 3.85 | 168.94 | 112.38 | 21.75 | 48.17 | 11.69 | 18.75 | 381.68 | retro pepsin |
| 3588:1-2136 | 100000 | 3.44 | 85.27 | 53.41 | 13.38 | 32.45 | 7.84 | 8.72 | 201.07 | unknown |
| 3625:1-1462 | 100000 | 3.72 | 114.26 | 67.26 | 12.88 | 29.95 | 8.66 | 13.62 | 246.63 | gag poly |
| 3705:307-2529 | 100000 | 3.96 | 116.19 | 67.04 | 12.48 | 31.56 | 7.49 | 9.84 | 244.6 | transposon |
| 2283:1-658 | 110000 | 3.66 | 37.48 | 33.68 | 5.47 | 12.87 | 2.83 | 3.19 | 95.52 | asparagine synthase |

**S2 Table**. Continued

| Unigene* | Boolean§ | Log  Ratio | MLDS | MHDS | MJBJ | TDBG | THBJ | TNRJ | Expression sum | BLASTX annotation |
| --- | --- | --- | --- | --- | --- | --- | --- | --- | --- | --- |
| 2593:1-346 | 110000 | 4.26 | 518.18 | 447.91 | 34.57 | 175.54 | 27.06 | 30.82 | 1234.08 | NBS-LRR class RGA |
| 2862:164-476 | 110000 | 4.07 | 127.14 | 81.59 | 13.54 | 43.87 | 7.55 | 14.28 | 287.97 | unknown |
| 3429:1925-2859 | 110000 | 4.37 | 540.02 | 431.62 | 54.73 | 184.66 | 26.06 | 43.79 | 1280.88 | retroelement |
| 3429:2882-4380 | 110000 | 4.27 | 122.57 | 87.94 | 11.94 | 37 | 6.35 | 9.77 | 275.57 | retro trans gag protein |
| 3519:104-811 | 110000 | 4.69 | 212.52 | 113.9 | 16.21 | 47.38 | 10.99 | 8.23 | 409.23 | unknown |
| 3692:16-469 | 110000 | 4.10 | 95.09 | 64.29 | 5.55 | 31.73 | 7.02 | 5.65 | 209.33 | unknown |
| 612:1-200 | 110000 | 4.14 | 97.5 | 76.57 | 10.21 | 36.98 | 5.54 | 10.1 | 236.9 | cDNA clone |
| 26295:48-773 | 110001 | 5.04 | 12.09 | 40.09 | 6.03 | 10.03 | 1.13 | 26.04 | 95.41 | chlorophyll a/b binding |
| 26303:1-186 | 110001 | 5.15 | 31.01 | 41.21 | 6.45 | 12.78 | 2.09 | 67.28 | 160.82 | chlorophyll a/b binding |
| 13975:550-819 | 110100 | 5.07 | 22.15 | 48.72 | 11.56 | 22.19 | 5.46 | 1.22 | 111.3 | RNA binding protein |
| 13984:660-931 | 110100 | 4.63 | 31.9 | 71.17 | 10.14 | 31.3 | 7.56 | 2.42 | 154.49 | RNA binding protein |
| 15100:417-718 | 110100 | 5.25 | 29.96 | 73.68 | 11.13 | 29.02 | 6.67 | 1.63 | 152.09 | RNA binding protein |
| 15153:443-725 | 110100 | 5.82 | 22.42 | 68.4 | 7.84 | 31.98 | 3.56 | 1.02 | 135.22 | RNA binding protein |
| 19930:2-269 | 110100 | 4.94 | 33.55 | 73.03 | 11.63 | 36.94 | 7.38 | 2 | 164.53 | unknown |
| 222:93-327 | 110100 | 6.02 | 59.63 | 35.35 | 1 | 10.89 | 0.92 | 3.74 | 111.53 | unknown |
| 2562:1-637 | 110100 | 5.46 | 68.6 | 39.23 | 8.21 | 21.98 | 1.56 | 3.87 | 143.45 | unknown |
| 3428:65-499 | 110100 | 4.83 | 183.27 | 338.22 | 69.68 | 202.94 | 52.07 | 11.92 | 858.1 | RNA recog motiff |
| 1192:213-508 | 111100 | 6.59 | 61.52 | 128.16 | 33.84 | 49.03 | 8.39 | 1.11 | 282.05 | unknown |
| 13985:600-903 | 111100 | 6.69 | 56.53 | 132.53 | 29.61 | 42.34 | 8.68 | 1.08 | 270.77 | RNA binding protein |
| 15148:554-761 | 111100 | 6.1 | 19.59 | 48.78 | 8.07 | 16.88 | 4.29 | 0.6 | 98.21 | RNA binding protein |
| 7068:1-292 | 111100 | 5.88 | 16.49 | 75.96 | 70.22 | 50.02 | 1.2 | 6.49 | 220.38 | heat shock protein |
| 9464:103-200 | 111101 | 5.82 | 8.1 | 24.12 | 18.98 | 21.21 | 0.39 | 14.72 | 87.52 | alpha-amylase inhibitor |
| 15093:558-772 | 111110 | 6.11 | 51.39 | 104.25 | 21.7 | 31.09 | 5.59 | 0.38 | 214.4 | RNA binding protein |
| 20213:1-297 | 111110 | 7.84 | 24.04 | 51.89 | 13.94 | 20.67 | 4.82 | 0.14 | 115.5 | unknown |
| 20525:1-242 | 111110 | 8.36 | 30.4 | 55.97 | 13.62 | 25.36 | 8.02 | 0.68 | 134.05 | unknown |
| 3480:1-254 | 111110 | 7.03 | 257.59 | 441.87 | 123.65 | 203.77 | 66.54 | 3.39 | 1096.81 | RNA binding protein |

*- cDNA unigene and the start and stop positions within the sequence alignment.

§- Boolean search terms. Numbers correspond to the columns of read counts. Log ratios are based on log2-((sum(1)/sum(0))
