# Supplementary material for: Wheat differential gene expression induced by different races of Puccinia triticina
Source: PLoS One. 2018 Jun 7;13(6):e0198350. doi: 10.1371/journal.pone.0198350 (PMC5991701; doi:10.1371/journal.pone.0198350)
Supplement: S3 Table — The primer number in the name column corresponds to the cDNA contig made from assembled wheat cDNAs. The “F” is the forward primer and the “R” is the reverse primer of the primer pair. (DOCX) [file pone.0198350.s003.docx]

**S3 Table. Primer sequences for real-time PCR primers**. The primer number in the name column corresponds to the cDNA contig made from assembled wheat cDNAs. The “F” is the forward primer and the “R” is the reverse primer of the primer pair.

| Primer Name | GenBank |  | Sequence | |  |
| --- | --- | --- | --- | --- | --- |
| 16780 F | JZ976969 | multiprotein bridging factor 1 (*Triticum aestivum*) ACO36694.1 | | TGGGATTGGTCCTATCAG | |
| 16780 R |  |  |  | CAGGTCACTTGAAACACG | |
| 959 F | JZ976970 | glycine rich RNA binding protein (*T. aestivum*) AGI04359.1 | | AACTGGAGGGAGTGAATG | |
| 959 R |  |  |  | AACATCTCGGAACCTACG | |
| 959 F |  |  |  | AAGTGTTATCGCTCGCCTC | |
| 959 R |  |  |  | TCATTCATAGCCAACGGG | |
| 13973 F | JZ976972 | glycine rich RNA binding protein (*T. aestivum*) AGI04359.1 | | CGGTAACAAGTAACACGG | |
| 13973 R |  |  |  | AACTGGAGGGAGTGAATG | |
| 1519 F | JZ976971 | glycine rich RNA binding protein (*T. aestivum*) AGI04359.1 | | AACTGGAGGGAGTGAATG | |
| 1519 R |  |  |  | AACAGGTAACACGGAACG | |
| 1519 F |  |  |  | AACAGGTAACACGGAACG | |
| 1519 R |  |  |  | AACATCTCGGAACCTACG | |
| 777 F | JZ976941 | glycine rich RNA binding protein (*T. aestivum*) AGI04359.1 | | AACTGGAGGGAGTGAATG | |
| 777 R |  |  |  | GACGGTAACAAGTAACACG | |
| 24701 F | JZ976947 | universal stress protein A like protein (*Aegilops tauschii*) EMT01474.1 | | ATCATAGACGAGTCAGCG | |
| 24701 R |  |  |  | ACCAGGAGTAGTTTGGAG | |
| 13975 F | JZ976964 | glycine rich RNA binding protein (*T. aestivum*) AGI04359.1 | | AACTGGAGGGAGTGAATG | |
| 13975 R |  |  |  | AACATCTCGGAACCTACG | |
| 13984 F | JZ976977 | glycine rich RNA binding protein (*T. aestivum*) AGI04359.1 | | AACTGGAGGGAGTGAATG | |
| 13984 R |  |  |  | AACATCTCGGAACCTACG | |
| 15100 F | JZ976965 | glycine rich RNA binding protein (*T. aestivum*) AGI04359.1 | | AACTGGAGGGAGTGAATG | |
| 15100 R |  |  |  | AACATCTCGGAACCTACG | |
| 15153 F | JZ976966 | glycine rich RNA binding protein (*T. aestivum*) AGI04359.1 | | TCTGGCTACCGTTAGATG | |
| 15153 R |  |  |  | CACATTAGGGACCACTTTG | |
| 19930 F | JZ976942 | glycine rich RNA binding protein (*T. aestivum*) AGI04359.1 | | AACATCTCGGAACCTACG | |
| 19930 R |  |  |  | AACTGGAGGGAGTGAATG | |
| 1192 F | JZ976943 | glycine rich RNA binding protein (*T. aestivum*) AGI04359.1 | | AACTGGAGGGAGTGAATG | |
| 1192 R |  |  |  | GAAGCATAGCGAACAGAG | |
| 13985 F | JZ976968 | glycine rich RNA binding protein (*T. aestivum*) AGI04359.1 | | AACTGGAGGGAGTGAATG | |
| 13985 R |  |  |  | ACATCTAACGGTAGCCTG | |
| 15148 F | JZ976976 | glycine rich RNA binding protein (*T. aestivum*) AGI04359.1 | | AACTGGAGGGAGTGAATG | |
| 15148 R |  |  |  | ACATCTAACGGTAGCCTG | |
| 20213 F | JZ976973 | glycine rich RNA binding protein (*T. aestivum*) AGI04359.1 | | GAAGCATAGCGAACAGAG | |
| 20213 R |  |  |  | AACTGGAGGGAGTGAATG | |
| 20525 F | JZ976959 | glycine rich RNA binding protein (*T. aestivum*) AGI04359.1 | | GAACAGTAACACGGAACG | |
| 20525 R |  |  |  | AATGGAGGGAGTGAATGC | |
| 12547 F | JZ976967 | type 1 non-specific lipid transfer protein precursor (*Triticum aestivum*) CAH69206.1 | | CTTATCTCCACAGGGTAAAC | |
| 12547 R |  |  |  | GGAACCACAAGAATCCTTAG | |
| 4596 F | JZ976948 | 4 hydroxphenylpyruvate dioxygenase (*Triticum aestivum*) AAZ67144.1 | | GCCGCATCTTACAAACAAC | |
| 4596 R |  |  |  | TCTTCACAAAGCCAGTGG | |
| 4231 F | JZ976962 | hypothetical protein (*Aegilops tauschii*) EMT24282.1 | | GTCCAACTGACTAACTGC | |
| 4231 R |  |  |  | CCTTGTGAGAACTATGAGG | |
| 3426 F | JZ976963 | chlorophyll a-b binding protein 1B-20, (*Triticum urartu*) EMS64136.1 | | ACAGCCTCCATTAGAAGC | |
| 3426 R |  |  |  | ATCATCCAGACCATCTCC | |
| 3428 F | JZ976975 | glycine rich RNA binding protein (*T. aestivum*) AGI04359.1 | | AACATCTCGGAACCTACG | |
| 3428 R |  |  |  | AACTGGAGGGAGTGAATG | |
| 955 F | JZ976940 | alanine-glyoxylate aminotransferase 2-like protein (*Aegilops tauschii*) EMT26999.1 | | CTTCGGCAATGTGTTCAG | |
| 955 R |  |  |  | ACAACGAGGTGCTGTTTC | |
| 222 F | JZ976949 | disease resistance protein RPM1 (*A. tauschii*) EMT03723.1, | | TCCCTTAGTGGAATCACGGC | |
| 222 R |  |  |  | AGTTCACACCCTTGCGGATG | |
| 4010 F | JZ976951 | pathogenesis related protein (*T. aestivum*) ACQ41879.1 | | CTCTGTGGCTTACAAGAATG | |
| 4010 R |  |  |  | GTACGGTACTCCTTGATTGA | |
| 2862 F | JZ976952 | hypothetical protein F775-10388 (*Aegilops tauschii*) EMT26923.1 | | CTTGTGGCTTCAGACTTCTA | |
| 2862 R |  |  |  | ATACATGTAGCCCATCAAGG | |
| 16104 F | JZ976953 | photosystem II reaction center protein Z (*Triticum urartu*) EMS61694.1 | | TACTAAGACTGAGGAAGTCG | |
| 16104 R |  |  |  | GAGGATTTGATGGTAGCTTG | |
| 16104 F |  |  |  | CCATCAGGAGAAGCAAATAC | |
| 16104 R |  |  |  | GGTAGCTTGGAGGATTAGAA | |
| 22994 F | JZ976954 | ER molecular chaperone (*T. aestivum*) AGN94841.1 | | GGCCAACAGACTAATAACAG | |
| 22994 R |  |  |  | CGAGGAGACATGGATAATTG | |
| 9694 F | JZ976955 | predicted protein (*Hordeum vulgare*) BAJ93722.1 | | GTGATATCTGCCAAATCGGA | |
| 9694 R |  |  |  | GAGTTCAGACCCATGCTTAG | |
| 7068 F | JZ976956 | heat shock protein (BiP protein) HSP70 (*Triticum aestivum*) AAB99745.1 | | ATGTTGTAGGCGTAGTTCTC | |
| 7068 R |  |  |  | CTGACAAGGAGGAGATTGAG | |
| 1911 F | JZ976957 | unnamed protein product (*Triticum aestivum*) CDM81404.1 | | CTACCAAACCAGCAAGTAAC | |
| 1911 R |  |  |  | GACCTCTGCTGAGAATAAGA | |
| 1911 F |  |  |  | GTAAGGTGTTGGGTTAAGTC | |
| 1911 R |  |  |  | ATAGGTACTCCCTCTCCTTC | |
| PR1 F |  |  | | CGGGAATATCATTGGACAGA | |
| PR1 R |  |  | | CGATTAGGGACGAAAGACTA | |
| PR2 F |  |  | | GGATGTTGCTTCCATGTTTG | |
| PR2 R |  |  | | ATGGATTGCACACTCATAGG | |
| PR5 F |  |  | | CTACCAGATCACCTTCTGTC | |
| PR5 R |  |  | | GCGGCTGTAATATGACAATG | |
| 16209 F | JZ976944 | ER molecular chaperone (*Triticum aestivum*) AGN94841.1 | | GCTGTTATTAGTCTGTTGGC | |
| 16209 R |  |  |  | GCATCATCTTTCCTTCATCC | |
| 16209 F |  |  |  | TTTCACCACCTACCAGGACC | |
| 16209 R |  |  |  | TTCAGGATACCGTTGGCGTC | |
| 2283 F | JZ976945 | glutamine-dependent asparagine synthetase (*Triticum aestivum*) AAU89392.1 | | CATTTGATTCTGCGTGAGC | |
| 2283 R |  |  |  | TTGATGACGAGGAGCAAC | |
| 24701 F | JZ976947 | universal stress protein A like protein (*Aegilops tauschii*) EMT01474.1 | | ATCATAGACGAGTCAGCGG | |
| 24701 R |  |  |  | ACGACCAGGAGTAGTTTGG | |
| 16208 F | JZ976939 | ER molecular chaperone (*Triticum aestivum*) AGN94841.1 | | TGACTTCGCTCTGAGGAGTG | |
| 16208 R |  |  |  | TCCAGGACTTTGAGGGTGAG | |
| 15083 F | JZ976958 | glycine rich RNA binding protein (*T. aestivum*) AGI04359.1 | | AACTGGAGGGAGTGAATG | |
| 15083 R |  |  |  | AACATCTCGGAACCTACG | |
| 3588 F | JZ976960 | glutathione-S-transferase 19E50 (*Triticum aestivum*) AAL47688.1 | | CTCCGCAAGTACAAGAAGAAC | |
| 3588 R |  |  |  | TTGATGAGGCACTCGTACAC | |
| 3318 F | JZ976961 | unnamed protein product (*Triticum aestivum*) CDM81404.1 | | CTCTTATTCTCAGCAGAGGT | |
| 3318 R |  |  |  | AAGACGAACGAATTGATGAC | |
| 20525 F | JZ976959 | glycine rich RNA binding protein (*T. aestivum*) AGI04359.1 | | CGGAGCGAGATCTAGGATAC | |
| 20525 R |  |  |  | GCAAATGGAGGGAGTGAATG | |
| 3692 F | JZ976978 | mRNA turnover 4-like protein (*Aegilops tauschii*) EMT02955.1 | | GAAAGAAGGTCATGCAGATAG | |
| 3692 R |  |  |  | CACCTCAAATTCTCGGAATAA | |
| 2593 F | JZ976979 | cytosolic malate dehyrogenase (*Triticum aestivum*) AAT64932.1 | | CCCTTCGAATGTGGATTTAC | |
| 2593 R |  |  |  | CCTGCTGGTCTTATCTACTC | |
| 612 F | JZ976980 | unknown | | AGAGAGGGAATTCTTGAGAT | |
| 612 R |  |  |  | CGTACATCAGGGAACTACTA | |
| 3503 F | JZ976981 | phosphoglycerate kinase (*Triticum urartu*) EMS54865. | | CATCAGGAGCAAACTTATCA | |
| 3503 R |  |  |  | GAGGGTTGAGGAAGATAAAC | |
| 38 F | JZ976982 | ananian (*Triticum urartu*) EMS66997.1 | | GTCAACAACAACATCCTCAC | |
| 38 R |  |  |  | CCCTCGCATATATACCTCTC | |
| 3429 F |  | RuBisCO (*Triticum aestivum*) BAB19814.1 | | TCGTACAAACACAGATCATAC | |
| 3429 R |  |  |  | GCCTAAACTACGAGTTGAAA | |
| 3429 F |  |  |  | ATCACACTGGTGTTGTATATG | |
| 3429 R |  |  |  | GGCCTATGTATGATTGTCTTT | |
| 23621 F |  | hypothetical protein (*Aegilops tauschii*) EMT31540.1 | | ACTTGATTGTCCTGGTTTAC | |
| 23621 R |  |  |  | GAAGATTGCAGATGTGATGA | |
| 23621 F |  |  |  | CCTGGTTTACTATCGGTAATCT | |
| 23621 R |  |  |  | TGATGACATCTCCAGCAATC | |
| 25668 F |  | cysteine proteinase inhibitor (*Triticum aestivum*) BAB18766.1 | | TTAAAGTTCTCCCATGGTTTC | |
| 25668 R |  |  |  | GCTGAAGAAGCAAATTGTTG | |
| 15606 F |  | chlrorplast ribulose-1,5-bisphosphase carboxylase activase (*Triticum aestivum*) ABI96906.1 | | AGAAGTACGACTTCGACAAC | |
| 15606 R |  |  |  | CCAGATACCCAAGATGAGTG | |
| 14659 F |  | ER molecular chaperone (*T. aestivum*) AGN94841.1 | | GGCCAACAGACTAATAACAG | |
| 14659 R |  |  |  | CTGTAGATGGTTTGGAAAGG | |
| 12547 F | JZ976967 | type 1 non-specific lipid transfer protein precursor (*Triticum aestivum*) CAH69206.1 | | GAATAATCTGAACGTGGTGTA | |
| 12547 R |  |  |  | CCTGAACATTAGTCTGTGTG | |
| 12574 F |  | hypothetical protein TRIUR3-31426 (*Triticum urartu*) EMS50722.1 | | GCGCTATTATATATGGTGGATT | |
| 12574 R |  |  |  | AGCTCGGATGAAGTTTAATG | |
| 15093 F | JZ976946 | glycine rich RNA binding protein (*T. aestivum*) AGI04359.1 | | CTGGCCAGTTATCCTAGTTAC | |
| 15093 R |  |  |  | CTGAACATCTCGGAACCTAC | |
| 2562 F |  | cytosolic malate dehyrogenase (*Triticum aestivum*) AAT64932 | | AAGGAATGGAAAGGAAGGAT | |
| 2562 R |  |  |  | CAGGGATAGATGAGCAAACT | |
| 3480 F | JZ976950 | ribulose-1,5-bisphosphate carboxylaase/oxygenase small subunit (*Triticum aestivum*) BAB19812.1 | | CAACTCTGACATTGCTTTGG | |
| 3480 R |  |  |  | CCGATAGCATTCACATACGA | |
| 3480 F |  |  |  | GAGGAGGTCAAGAAGGAGTA | |
| 3480 R |  |  |  | CCAAAGCAATGTCAGAGTTG | |
| 15093 F | JZ976946 | glycine rich RNA binding protein (*T. aestivum*) AGI04359.1 | | AACTGGAGGGAGTGAATGG | |
| 15093 R |  |  |  | AACATCTCGGAACCTACGC | |
| 3480 F | JZ976950 | ribulose-1,5-bisphosphate carboxylaase/oxygenase small subunit (*Triticum aestivum*) BAB19812.1 | | ACTGGAGGGAGTGAATGGTG | |
| 3480 R |  |  |  | AGCCTGAACATCTCGGAACC | |
| Ubq F |  |  | | GCACCTTGGCGGACTACAACATTC | |
| Ubq R |  |  | | GACACCGAAGACGAGACTTGTGAACC | |
